# Supplementary material for: Evaluation framework study assessing the role, applicability and adherence to good practice of planning support tools for allocation of development aid for health in low-income and middle-income countries
Source: BMJ Open. 2023 Jul 12;13(7):e069590. doi: 10.1136/bmjopen-2022-069590 (PMC10347503; doi:10.1136/bmjopen-2022-069590)
Supplement: Supplementary data [file bmjopen-2022-069590supp001.pdf]

Supplementary Material 1. Tool documentation

CAPACITI (Country-led Assessment for Prioritization in Immunization)
Guidance manual

PriorityVax
User guide
Phase 1 report
Phase 2 report
Phase 3 report

Optima
HIV User Guide
TB User Guide
HIV allocative efficiency analysis training workshop agenda and training materials
TB allocative efficiency analysis training workshop agenda and training materials
Software Reference Manual
Expenditure Data Guide
HIV-related Abbreviations
Parameter Data Sources

OneHealth
FAQ
Startup Manual
Technical Notes
Technical Notes Annexes
Intervention Input Assumptions Manual
Defining the target population PIN and coverage in OneHealth
Using OneHealth for programme planning
MNS Module Manual
Manual

LiST (Lives Saved Tool)
Manual
Subnational modeling
Nutrition modeling in LiST

EQUIST (Equitable strategies to save lives)
Technical notes
User guide

Supplementary materials 2. Tables

Table 1. Tools additional information

	Year created	Owned / developed by	Time/process to implement	Required level of technical expertise
CAPACITI	2020	WHO	A typical recommendation is expected to take from 4 to 6 months. Intensive data collection required.<br><br>1. Clarify decision question<br>2. Set criteria for decision-making<br>3. Collect and assess evidence<br>4. Appraisal of results<br>5. Finalisation and communication of recommendation	Minimal (No/minimal training is required)
PriorityVax	2010**	Sabin Vaccine Institute	No information given on timing of process.<br><br>1. Policy questions are defined (Define decision criteria, Discuss and identify attributes, Explicitly weight criteria, Discuss and weight attributes)<br>2. PICO questions are defined (Population, Intervention, Comparator, Outcome)<br>3. Evidence gathered and reviewed to address PICO questions and evidence attributes<br>4. Evidence used to extract values and/or score attributes<br>5. Recommendation provided<br>6. Interactive sensitivity analysis	Minimal (No/minimal training is required)
HIPtool	NA	WB	No formal information on process, intensive data collection required.	Minimal (No/minimal training is required)
Optima	2004	OPTIMA consortium	No formal information on process, intensive data collection required.	Detailed knowledge required (3 days intensive training)
OneHealth (Spectrum suite)	1997	Avenir Health	A typical recommendation is expected to take from 4 to 6 months:<br><br>1. Initiation of planning process.<br>2. Stakeholder meeting (first plenary).<br>3. Tool set-up with baseline data entry.<br>4. Second plenary.<br>5. Individual modules/groups.<br>6. Assessing the joint plan.<br>7. Adjusting individual modules/groups.<br>8. Submission of revised modules.<br>9. Group discussion and final presentation of NHSP (National health service plan)	Detailed knowledge required
LiST	2003	Institute for International Programs at Johns Hopkins Bloomberg School of Public Health	No information in user manual or technical notes on likely process and timelines for implementation	Minimal (No/minimal training is required)
EQUIST	2016	UNICEF in association with Johns Hopkins Bloomberg School of Public Health and WB	No information given on timing of process.<br><br>Users conduct a situational analysis, comprising an equity profile analysis and an equity frontier analysis, followed by a scenario analysis, as follows:<br><br>1. Define priority / target populations<br>2. Prioritise epidemiological priorities<br>3. Prioritise interventions<br>4. Prioritise key bottlenecks	Minimal (No/minimal training is required)

			5. Prioritise causes of bottlenecks<br>6. Select strategies to address cause of bottleneck<br>7. Assess expected impact and cost effectiveness	

CAPACITI: Country-led Assessment for Priortization in Immunization; HIPtool: Health Interventions Priortization tool; LiST: Lives Saved Tool; EQUIST: Equitable strategies to save lives; LMIC: Low- and Middle-Income Country; MNCH&N: Maternal, Newborn, Child Health and Nutrition.

* Adaptability to extend to new diseases and interventions.

** The Institute of Medicine commissioned the tool in 2010, and it was developed with the name SmartVaccines. This evolved into PriorityVax by 2018.
