# Supplementary material for: Evaluation framework study assessing the role, applicability and adherence to good practice of planning support tools for allocation of development aid for health in low-income and middle-income countries
Source: BMJ Open. 2023 Jul 12;13(7):e069590. doi: 10.1136/bmjopen-2022-069590 (PMC10347503; doi:10.1136/bmjopen-2022-069590)
Supplement: Author's manuscript [file bmjopen-2022-069590.draft_revisions.pdf]

BMJ Open is committed to open peer review. As part of this commitment we make the peer review history of every article we publish publicly available.

When an article is published we post the peer reviewers' comments and the authors' responses online. We also post the versions of the paper that were used during peer review. These are the versions that the peer review comments apply to.

The versions of the paper that follow are the versions that were submitted during the peer review process. They are not the versions of record or the final published versions. They should not be cited or distributed as the published version of this manuscript.

BMJ Open is an open access journal and the full, final, typeset and author-corrected version of record of the manuscript is available on our site with no access controls, subscription charges or pay-per-view fees (http://bmjopen.bmj.com).

If you have any questions on BMJ Open's open peer review process please email info.bmjopen@bmj.com

BMJ Open

The role and applicability of planning support tools for allocation of development aid for health

Journal:	*BMJ Open*
Manuscript ID	bmjopen-2022-069590
Article Type:	Original research
Date Submitted by the Author:	01-Nov-2022
Complete List of Authors:	Megiddo, Itamar; University of Strathclyde, Department of Management Science<br>Blair, Shona; University of Strathclyde, Department of Management Science<br>Sabei, Davood; University of Strathclyde, Department of Management Science<br>Ruiz, Francis; London School of Hygiene & Tropical Medicine, Department of Global Health and Development; Center for Global Development<br>Morton, Alexander; University of Strathclyde
Keywords:	Health economics < HEALTH SERVICES ADMINISTRATION & MANAGEMENT, Health policy < HEALTH SERVICES ADMINISTRATION & MANAGEMENT, International health services < HEALTH SERVICES ADMINISTRATION & MANAGEMENT

SCHOLARONE™
Manuscripts

I, the Submitting Author has the right to grant and does grant on behalf of all authors of the Work (as defined in the below author licence), an exclusive licence and/or a non-exclusive licence for contributions from authors who are: i) UK Crown employees; ii) where BMJ has agreed a CC-BY licence shall apply, and/or iii) in accordance with the terms applicable for US Federal Government officers or employees acting as part of their official duties; on a worldwide, perpetual, irrevocable, royalty-free basis to BMJ Publishing Group Ltd ("BMJ") its licensees and where the relevant Journal is co-owned by BMJ to the co-owners of the Journal, to publish the Work in this journal and any other BMJ products and to exploit all rights, as set out in our licence.

The Submitting Author accepts and understands that any supply made under these terms is made by BMJ to the Submitting Author unless you are acting as an employee on behalf of your employer or a postgraduate student of an affiliated institution which is paying any applicable article publishing charge ("APC") for Open Access articles. Where the Submitting Author wishes to make the Work available on an Open Access basis (and intends to pay the relevant APC), the terms of reuse of such Open Access shall be governed by a Creative Commons licence – details of these licences and which Creative Commons licence will apply to this Work are set out in our licence referred to above.

Other than as permitted in any relevant BMJ Author's Self Archiving Policies, I confirm this Work has not been accepted for publication elsewhere, is not being considered for publication elsewhere and does not duplicate material already published. I confirm all authors consent to publication of this Work and authorise the granting of this licence.

For peer review only

The role and applicability of planning support tools for allocation of development aid for health

Itamar Megiddo, PhD[1,*], Shona Blair, PhD[1], Davood Sabei, PhD[1], Francis Ruiz, MSc[2,3], Alec Morton, PhD[1]

[1] Department of Management Science, University of Strathclyde, Glasgow, UK
[2] Department of Global Health and Development, London School of Hygiene and Tropical Medicine, London, UK.
[3] Center for Global Development Europe, Washington, London, UK.

Dr Itamar Megiddo
Department of Management Science
University of Strathclyde
199 Cathedral Street
Glasgow, G4 0QU
Email: itamar.megiddo@strath.ac.uk
Phone: 44 (0)141 548 3452

Key words: development aid for health (DAH); health planning tools; prioritization.

For peer review only

Abstract

Objectives: Allocation of Development Aid for Health (DAH) is controversial and challenging. In recent years, several planning-software tools have promised to help decision-makers align resource allocation with their objectives, more clearly connect prioritisation to evidence and local circumstances, and increase transparency and comparability. We aim to explore these tools to provide insight into their fitness for purpose and suggest future directions to fulfil that promise.

Methods: We identified seven tools that met the inclusion criteria. Our evaluation framework compares them along two dimensions: ability to produce analyses adhering to principles laid out in the International Decisions Support Initiative (iDSI) Reference Case for health economic evaluations; and resources required, including expertise and time. We extracted information from documentation and tool use and sent this information to tool developers for confirmation.

Results: We categorise the tools into evidence-generating ones, evidence-syntheses ones, and process support ones. Tools' fitness for purpose varies by the context, technical capacity, and time limitation. The tools adhere to several Reference Case principles but often not to all of them. The source and underlying assumptions of pre-populated data are often opaque. Comparing vertical interventions across diseases and health system strengthening ones remains challenging.

Conclusions: The plethora of tools that aid priority setting in different ways is encouraging. Developers and users should place further emphasis on their ability to produce analyses that

For peer review only - http://bmjopen.bmj.com/site/about/guidelines.xhtml

adhere to prioritisation principles. Opportunities for further development include using evidence-generating tools and Multi-Criteria Decision Analysis approaches complimentarily. However, maintaining tool simplicity should also be considered to allow wider access.

For peer review only

For peer review only - http://bmjopen.bmj.com/site/about/guidelines.xhtml

Key messages

What is already known about the topic: Allocation of Development Aid for Health (DAH) planning tools promise to connect resource allocation to evidence and local circumstances in a transparent and rigorous manner while creating a shared understanding among stakeholders.

What this study adds: The study categorises the tools into three groups that help define the context in which they are useful for purpose: evidence-generating tools, evidence-syntheses tools, and process support tools. The tools balance increasing access—by simplifying analyses and time required—and flexibility to develop context-specific analyses. In some cases, restrictions that simplify analysis increase difficulty to adhere to good principles of prioritisations.

How this study might affect research, practice or policy: Future development could improve tools ability to compare vertical, disease-specific interventions with health strengthening ones. Additionally, stakeholders and analysts can use quantitative evidence-generating tools and process support tools such as multi-criteria approaches complimentarily and developers have the opportunity to integrate them.

For peer review only

For peer review only - http://bmjopen.bmj.com/site/about/guidelines.xhtml

Introduction

National health planning tools that simplify the process of priority setting have the potential to aid in adhering to the guiding principles of prioritisation. Allocation of development aid for health (DAH) is always controversial and challenging, and the emergence of COVID-19 as a fourth major internationally endemic communicable disease exacerbates the difficulty of overcoming these challenges in a more constrained financial environment. The guiding principles suggest that prioritisation should transparently reflect the values of the country—represented through stakeholders—and this requires a shared understanding across stakeholders of the prioritisation process and the evidence used in this process.[1] Gaining this shared understanding may be difficult due to the differing knowledge base among stakeholders on health interventions and technical methods for evaluating them. In recent years, research groups and global health and development organisations have developed tools for national health planning in low- and middle-income countries (LMICs). These tools simplify comparison across programmes and can inform the discussion in the prioritisation process and help develop an evidence base.

It is important to realise that tools support rather than make decisions. A key insight from Health Technology Assessment experience in high-income countries is that effective priority setting requires both technical methods and tools on the one hand and institutions and institutional processes on the other.[2] A number of different process frameworks have been advocated for prioritisation decisions: Socio-Technical Allocation of Resources (STAR),[3] Program Budgeting and Marginal Analysis,[4] Accountability for Reasonableness,[5] and Evidence-Informed Deliberative Process.[6] Though tools and the processes they support are often difficult to separate, in this paper we are agnostic about how the prioritisation process should be structured. We define a *national health planning tool* as a software package that

For peer review only - http://bmjopen.bmj.com/site/about/guidelines.xhtml

requires input and produces outputs specifically designed to support its users in planning. In the rest of the paper, we restrict our analysis and discussion to tools that provide a quantitative analysis, but their purpose may be to generate evidence for use as part of the prioritisation process or to support the process in other ways. Our focus is on these tools applicability and ability to support or produce analyses that are useable within the context of some structured prioritisation process.

Tool developers make several choices that influence the tools' applicability in these contexts, including ones affecting the required level of expertise to use them and users' ability to adhere to good practice. Tools can offer users more or less flexibility, though they all limit this flexibility to some extent: unlimited flexibility does away with the tool and leaves all decisions up to the analysts conducting the prioritisation exercise. This flexibility has benefits such as improving the analysts' ability to utilise the most novel methods and not restricting contextualisation (beyond data availability limitations). However, it also reduces access to conducting prioritisation to those with very high levels of expertise and increases the time costs of a given exercise. The choices made by tool developers limit that flexibility while defining the inherent 'tool opinion' about what ingredients are important for its given purpose. Choices that restrict specific inputs (e.g., the number of years into the future considered) may limit users' ability to adhere to certain prioritisation guiding principles, while in other cases, they can guide or steer—and in some cases force—users to adhere to certain principles, exhibiting that 'tool opinion'.

Further, the choices made on the presentation of tools' outputs affect accessibility for users of these outputs. The sheer range of stakeholders that contribute to priority setting can lead to demands for different types of outputs. Their differing knowledge base can contribute to

For peer review only - http://bmjopen.bmj.com/site/about/guidelines.xhtml

information asymmetries in the prioritisation process and the evidence it uses. The Global Fund to Fight Aids, Tuberculosis, and Malaria (GFATM), for example, utilises country coordinating mechanisms (CCMs) to submit applications and govern its DAH funds. These CCMs are responsible for empowering and representing the views of stakeholders from across sectors (e.g., academic institutions, civil society, government, those with lived experience, NGOs, and private institutions), and they are asked to submit an application for funding that includes a description of their prioritisation process.[7] A tool with output that simplifies the understanding of evidence or improves the process to incorporate the disparate voices would go a long way toward achieving the vision for CCMs. However, asking tools to cater to all of these stakeholders as well as to donor agendas—that often require information on impact, value for money, and equity—is a tall order.

The global health community has a limited grasp on the success of these tools' fulfilment of the promise to increase access to conducting and utilising the outputs of prioritisation exercises in a way that is consistent with the principles of good prioritisation. This gap makes it difficult to decide on the context in which one tool should be used rather than another. The World Bank has explored tools for allocative efficiency within the context of HIV, explaining the purpose of a few tools and broadly discussing some of their limitations. The literature on good principles for infectious disease and health economic modelling in LMICs and communication of these models is more mature.[1,8] A recent study, compared COVID-19 models along dimensions of model characteristics and reporting.[9,10] Based on these elements, the study defined a framework for thinking about the models' fitness for purpose, setting questions to guide both output users—policymakers in this case—and analysts that adapt these models to contextualise an analysis.[9] Both of these audiences are also important for characterising *national health planning tools* that support priority setting.

For peer review only - http://bmjopen.bmj.com/site/about/guidelines.xhtml

Our paper aims to provide an understanding of these tools' characteristics that influence their suitability for a given purpose and gain an insight into future directions for these tools. We develop an evaluation framework that considers the tools' ability to produce analyses or processes that adhere to good practice and the onus they put on their users in terms of expertise and time. We explore the availability of transparent documentation on the tools and the ease of navigating this documentation. We further describe the types of outputs they produce. We *do not* rank the tools or suggest that any are inadequate for some part of the priority setting process. In the following sections, we describe our methodology for developing and utilising our evaluation framework, describe our findings, and discuss conclusions and recommendations.

Methods

Tool selection

We selected planning software tools that aid in resource allocation based on the following inclusion criteria: the software package i) provides a quantitative analysis, ii) is applicable for multiple countries, iii) can be adapted to utilise new data, and iv) is available in the public domain during the evaluation period.

We identified seven tools that met our criteria through discussions with project partners and targeted literature searches: OneHealth Tool in combination with the Spectrum suite, the Lives Saved Tool (LiST), Optima, Health Interventions Prioritisation (HIP) tool, EQUitable Strategies to Save Lives Tool (EQUIST), PriorityVax, Country-led Assessment for Prioritisation on Immunization (CAPACITI) decision-support tool. LiST is part of the Spectrum suite, but we include it separately as it has been used standalone for many analyses. We excluded two tools we found in our initial search as they did not meet inclusion criteria

For peer review only - http://bmjopen.bmj.com/site/about/guidelines.xhtml

(i) and (iv); STAR and Cascade respectively. STAR is a process framework for allocation and does not include a tool that provides quantitative outputs. Cascade is an online tool, and its website was down when the analysis was conducted.

Development of evaluation framework

The evaluation framework is based on good practices of economic analyses as outlined by the iDSI Reference Case[1] as well as on the requirements for utilising the tools. The reference case lists eleven principles for economic evaluations (Table 1), and these were adapted as relevant for the resource allocation tools and organized in a set of four topics (with corresponding tables): modelling assumptions, model inputs, model outputs, and validity and transparency. Two additional topics (and corresponding tables) that do not correspond to the reference case principles provide an overview of the tools and describe the resources—in time and expertise—required for using the tools and understanding their outputs. Table 1 describes the corresponding principles for each evaluation table.

Table 1. Evaluation framework and the iDSI Reference Case Principles

Evaluation Framework Table	Principles
Tool overview and purpose	Does not correspond to principles
Resources required for analysis	Does not correspond to principles
Modelling assumptions	Principle 6 – Time horizon and discounting
	Principle 7 – The analysis perspective (non-health effects and costs outside the health budget should be included)
Inputs	Principle 3 – Systematic and comprehensive evidence gathering
	Principle 5 – Costs reflect all resources used
	Principle 8 – Heterogeneity in population subgroups should be explored
Outputs	Principle 4 – Appropriate measures of outcome
	Principle 8 – Heterogeneity in population subgroups should be explored
	Principle 9 – Uncertainty

For peer review only - http://bmjopen.bmj.com/site/about/guidelines.xhtml

	Principle 10 – Impact on budget and other constraints identified
	Principle 11 – Explore equity
Transparency and validity	Principle 1 – Transparency

We examined whether the tools allow users to adhere to these principles, as violating them in a particular prioritisation exercise will also depend on how the tools are used. We dropped Principle 2—the comparator accurately reflects the decision problem—which was particularly difficult to capture without considering the tool users' decisions. We do discuss the limitations tools adaptability to extensions that consider additional diseases and interventions (Tools overview and purpose), and this should provide sufficient information on the limitations of the comparators the user can evaluate.

Each table consists of items used in the data extraction process. Items do not evaluate appropriateness or adherence to the principles. Rather, they report information about the tools characteristics that can be used to evaluate adherence to these principles as well as the level of knowledge needed to use them.

Data extraction

Two members of the research team extracted information for the seven tools we evaluated on the items identified for each table in the evaluation framework. They experimented with the tools and extracted information based on documentation and publications on the tool. To assess usability, they explored tool documentation and, where available, identified the level of user expertise developers assumed or suggested. They then developed implemented rudimentary analyses using the tools' interfaces to understand how quickly they could set up an analysis after reading the documentation. After this extraction from documents and

For peer review only

experiments with the tools, the entire research team then discussed the data in the evaluation tables to develop the analysis.

Patient and public involvement

Patients and the public were not involved in this study.

Results

Tools overview and purpose

The tools can be categorised along different dimensions that help identify their role in a prioritisation exercise, including by their aims and diseases and interventions they include in an analysis (Table 2). Apart from EQUIST and Optima, the tools are not limited by diseases they can analyse.

Table 2. Tools overview

	Stated aim(s) of tool	Approach & theoretical foundations	Adaptability to extend (e.g., custom interventions)
CAPACITI	Support evaluation of immunisation options incorporating values and programme context	MCDA embedded in broader problem-structuring framework	Focus on immunisation programmes but can extend to other
PriorityVax	Support vaccine prioritisation; clarify data uncertainty impact; facilitate discussion; clarify stakeholder views	MCDA embedded in broader problem-structuring framework	Focus on immunisation programmes but can extend to toher
HIPtool	Support health intervention prioritisation at country level	Evidence-synthesis	Yes
Optima	Analyse and project HIV and TB epidemics; determine optimal resource allocations	Impact model: dynamic compartmental models; Costing: logistic cost function model; Global optimization to minimize outcome (e.g., DALYs) or costs to achieve defined outcomes	No

For peer review only - http://bmjopen.bmj.com/site/about/guidelines.xhtml

OneHealth (Spectrum suite)	Support to inform national health care planning and resource needs at country level, strengthening health system analysis, costing, and finance	Impact model: module specific (e.g., LiST for child and maternal health interventions, or TIME a dynamic compartmental model for TB); Costing: ingredients-based costing	Custom interventions can be added
LiST	Estimate the impact of scaling up MNCH&N interventions in LMICs	Impact model: static equation-based model; costing: ingredient-based costing	Focus on MNCH&N; Custom interventions can be added; only LMICs
EQUIST	Estimate the impact of scaling up MNCH&N interventions in LMICs	Bottleneck analysis and statistical analysis of changes to effective coverage; Impact analysis: based on LiST; Costing: based on LiST and OneHealth	Focus on MNCH&N; only LMICs

CAPACITI: Country-led Assessment for Priortization in Immunization; HIPtool: Health Interventions Priortization tool; LiST: Lives Saved Tool; EQUIST: Equitable strategies to save lives; LMIC: Low- and Middle-Income Country; MNCH&N: Maternal, Newborn, Child Health and Nutrition.
* Adaptability to extend to new diseases and interventions.

Further exploring the approaches and methods the tools use, we can categorize them into ones that generate new data (e.g., through impact and costing models), ones that synthesize existing data, and ones that focus on problem structuring and process (Table 2). Tools that generate new data produce predictions of intervention impact using simulation models. The HIPtool synthesizes evidence, relying on evidence generated in external studies. Process tools provide a broader problem-structuring framework such as multi-criteria decision analysis (MCDA; e.g., PriorityVax). They similarly rely on evidence generated in external studies, but their focus is on the process of the decision-making exercise. Different elements of EQUIST lie in different categories: the tool synthesises evidence to display outputs on bottlenecks and intervention prioritisation; and it guides users on process in a seven-step approach that includes stakeholder discussion.

For peer review only - http://bmjopen.bmj.com/site/about/guidelines.xhtml

Adherence to reference case methodological principles

The flexibility the tools provide users (e.g., to conduct analyses from different perspectives), their built-in assumptions, and the directions they steer users have implications for users' ability to develop analyses that adhere to the iDSI Reference Case principles (Table 3). The principles suggest analyses report a disaggregated societal perspective. Several tools allow users to conduct evaluations from different perspectives. Other tools focus on the health system perspective. A few of the tools restrict the time horizon to 2050, limiting the possibility to consider a lifetime horizon that is recommended by the principles. In evidence-synthesis and process-focused tools, the time horizon depends on the evidence they incorporate and the transparency and accessibility the tool provides users to its sources (if users use pre-populated data). Most tools allow users to conduct parameter sensitivity and uncertainty analyses. They do not consider stochastic uncertainty—random variation in outcomes—or uncertainty in the analysis structure.

Table 3. Modelling Assumptions

	Theoretical basis	Perspective	Time horizon	Forms of uncertainty explicitly considered*
CAPACITI	Multi-criteria decision analysis (MCDA) embedded in broader problem-structuring framework	Flexible	N/A	Parameter; Criteria weighting
PriorityVax	Multi-criteria decision analysis (MCDA) embedded in broader problem-structuring framework	Flexible	N/A	Parameter; Criteria weighting
HIPtool	Synthesise evidence	Health system	N/A	
Optima	Impact model: dynamic compartmental models; costing: logistic cost function model; global	Health system	Do not know	Parameter

For peer review only - http://bmjopen.bmj.com/site/about/guidelines.xhtml

	optimization to minimize outcome (e.g., DALYs) or costs to achieve defined outcomes			
OneHealth (Spectrum suite)	Impact model: module specific (e.g., LiST for child and maternal health interventions, or TIME a dynamic compartmental model for TB); costing: ingredients-based costing	Flexible	100 years fixed time horizon	Parameter
LiST	Impact model: static equation-based model; costing: ingredient-based costing	Flexible	Maximum until 2050	Parameter
EQUIST	Bottleneck analysis and statistical analysis of changes to effective coverage based on strategies' bottleneck reduction; impact analysis: based on LiST; costing: based on LiST and OneHealth	Health System	Maximum until 2050	

CAPACITI: Country-led Assessment for Priortization in Immunization; HIPtool: Health Interventions Priortization tool; LiST: Lives Saved Tool; EQUIST: Equitable strategies to save lives.
* The two forms of uncertainty in the table include: (i) Parameter – uncertainty in tool input values; (ii) Criteria weighting – uncertainty in the relative importance of criteria (MCDA specific). Users can explore different scenarios using the tools, including EQUIST, which can be used to assess uncertainty. As this feature is not automated but requires developing new analyses we do not include it in the table. Other forms of uncertainty such as model structural uncertainty are not explicitly considered by the tool and are thus not included.

Resource and data requirement

The required technical expertise and time investment vary with tool complexity (Table S2.1 in Supplementary Materials 2). Relatively novice users can implement analyses in tools that merely synthesise information. Though, the users do require an understanding of health economic evaluation terminology. Users need knowledge across a range of modules for more complex tools such as OneHealth and Optima. The tools do not consistently provide guidelines on the time an analysis requires. While tools such as CAPACITI and OneHealth recommend an analysis over four to six months, HIPtool does not require significant time investment if analysts and stakeholders use its pre-populated data.

Most tools require intensive data collection to conduct a contextualised analysis, albeit most, incorporate pre-populated data (Table 4). Where they do not provide pre-populated data, they

For peer review only - http://bmjopen.bmj.com/site/about/guidelines.xhtml

provide guidance on data collection and discuss sources in their literature.[11] The level of guidance varies by tool. Many of the tools reference commonly known global health data sources (e.g., the World Health Organization and the Global Burden of Disease [GBD] study). Additionally, they reference specific studies (e.g., OPTIMA).[12] We observed differing transparency levels about tools' data sources. PriorityVax, for example, provided a thorough discussion of data sources to support analysis. HIPtool noted its use of the GBD, Disease Control Priorities (DCP3) Essential Universal Health Coverage package and Highest-Priority Package data and "other secondary sources." The tool and its documentation do not provide the specific source and underlying assumptions of pre-populated data such as incremental cost-effectiveness ratios (ICERs).

Table 4. Data requirements

	Target population	Epidemiological	Intervention coverage	Intervention cost*	Preloaded data
CAPACITI	Flexible	Flexible	NA	Flexible	No
PriorityVax	Flexible	Flexible	NA	Flexible	Upon request
HIPtool	NA	Burden prevalence	Not an input; optimises investment	Intervention unit costs	Yes
Optima	By subpopulation (demographic, geography, and risk-group) and population dynamics**	Several inputs	Over time	Intervention unit costs	Yes (sources in documentation)
OneHealth (Spectrum suite)	By subpopulation (socio-demographic and geography)***;	Several inputs (varies by module)	Over time	Intervention; human-resources, logistics, and	Yes (sources in tool)

For peer review only - http://bmjopen.bmj.com/site/about/guidelines.xhtml

	population dynamics **			infrastructure for delivery	
LiST	By subpopulation (socio-demographic and geography); population dynamics**	Incidence, prevalence, and health indicators, depending on disease	Over time	Intervention; human-resources, logistics, and infrastructure for delivery	Yes (sources in tool)
EQUIST	By subpopulations (socio-demographic and geography)	Several inputs	Bottlenecks to effective coverage and their contribution	Yes	Yes (sources in tool); several inputs cannot be updated by the user but can be managed by the "Country administrator"

CAPACITI: Country-led Assessment for Priortization in Immunization; HIPtool: Health Interventions Priortization tool; LiST: Lives Saved Tool; EQUIST: Equitable strategies to save lives.
NA – not applicable; several of the categories are not applicable for evidence-synthesis and/or process tools such multi-criteria decision analysis (MCDA) ones; Flexible – as MCDA tools define criteria as part of the process, these inputs are flexible
* Users can combine costs to include them under one category; we note how tools disaggregate the cost data further (e.g., human resources and logistics).
** Population dynamic parameters include ones related to fertility and mortality (exogenous to diseases and interventions evaluated).
** The types of data included may vary by module within OneHealth.

The types of data tools require depends on the tools' aims (Table 4). For example, tools that estimate impact require detailed population and epidemiological data. EQUIST, which incorporates a bottleneck analysis, inputs include information about bottlenecks to effective coverage. The MCDA-based tools data requirements depend on the criteria decision-makers and stakeholders deem important. These tools can also include inputs such as ICERs, which tools that generate new data often consider as outputs. HIPtool, similarly, considers ICERs as an input. The HIPtool also requires equity and financial risk protection scores (and provides pre-populated values).

For peer review only - http://bmjopen.bmj.com/site/about/guidelines.xhtml

Outputs

Most tools use visualisation to present outputs on cost, health outcomes (or disease burden), and economic efficiency, with MCDA tools also including aggregated scores (Table 5). Both the HIPtool and Optima outputs include a comparison between baseline and optimal scenarios in terms of economic efficiency.

Table 5. Outputs

	Cost	Health outcomes	Economic efficiency	Type(s) of output	Uncertainty representation	Outputs can be disaggregated into subpopulations
CAPACITI	Yes	Yes	Yes	Aggregate scores, Charts, Tables	No	No
PriorityVax	Yes	Yes	Yes	Aggregate scores, Charts, Tables	No	Scores based on entire population
HIPtool	Yes	Yes	Yes, including optimal package of interventions	Charts	No	No
Optima	Yes	Yes	Yes, including optimal package of interventions	Charts, Tables	Yes, plots with parameter scenarios	Demographic and geospatial characteristics

For peer review only - http://bmjopen.bmj.com/site/about/guidelines.xhtml

OneHealth (Spectrum suite)	Yes	Yes	Yes	Charts, Tables	Yes, uncertainty bounds	Demographic characteristics
LiST	Yes	Yes	Yes	Charts, Tables	Yes, uncertainty bounds	Demographic characteristics
EQUIST	Yes	Yes	Yes	Charts, Tables	No	Demographic charecteristics, wealth, and geography

CAPACITI: Country-led Assessment for Priortization in Immunization; HIPtool: Health Interventions Priortization tool; LiST: Lives Saved Tool; EQUIST: Equitable strategies to save lives.

Only some of the tools present uncertainty in their outputs (Table 5). Uncertainty bounds are the most common representation of uncertainty among these tools. While the MCDA tools outputs do not to explicitly describe uncertainty in results, they recommend users conduct a sensitivity analysis to understand variation due to stakeholder preferences and criteria values uncertainty.

Several, but not all, tools allow users to disaggregate results by different demographic dimensions (Table 5). While MCDA tools do not present disaggregated outputs, they can include subpopulation outcomes as criteria. EQUIST disaggregates some outputs by wealth. The HIPtool does not produce disaggregated outputs.

Transparency

The majority of the tools provided some degree of documentation; however, we found variation in the accessibility of the documentation (Table 6). All tools have produced at least one analysis that was published in a peer reviewed journal. Frequently, such publications were written by the tool developers themselves, although not in all cases. Given the variety of

For peer review only - http://bmjopen.bmj.com/site/about/guidelines.xhtml

release dates of the tools (Table S2.1 in the Supplementary Materials), the lack of independent tool usage does not necessarily imply a lack of tool usage by other groups.

Table 6. Transparency and Validity

	Documentation availability*	Limitations explained	Interests of authors declared	Funding declared	Open source	Peer reviewed publications
CAPACITI	Yes	Yes, but no dedicated limitations section	No	No	Yes	Yes
PriorityVax	Yes	Yes, but no dedicated limitations section	No	Yes	No	Yes
HIPtool	No (work in progress)	No	No	No	No	Yes
Optima	Yes	Yes	Yes	Yes	No	Yes
OneHealth (Spectrum suite)	Yes	Yes	Yes	Yes	No	Yes
LiST	Yes	Yes	Yes	Yes	No	Yes
EQUIST	Yes	Yes, but no dedicated limitations section	No	Yes	No	Yes

CAPACITI: Country-led Assessment for Priortization in Immunization; HIPtool: Health Interventions Priortization tool; LiST: Lives Saved Tool; EQUIST: Equitable strategies to save lives.
* This included both guidance manuals and technical reports. Other forms of documentation (presentations, online discussions, secondary reports) were available for some tools; however, these have not been included in the evaluation.

For peer review only - http://bmjopen.bmj.com/site/about/guidelines.xhtml

Discussion and conclusion

The promise of health planning tools is that they will simplify the process of prioritising health interventions and resource allocation in a manner that consistently adheres to accepted principles while also creating trust and a shared understanding among stakeholders. In this study, we developed and implemented an evaluation framework to describe tool characteristics that convey where the tools succeed and provide insight into potential future directions for tool developers. Our hope is that comparing tool characteristics also helps tool users identify which tool—or tools—are well suited for their prioritisation exercise.

Based on our findings on tool purpose and the types of inputs they require, we group the tools into three nonexclusive categories: evidence-generating, evidence-synthesising, and process. Evidence-generating tools produce outputs used in the prioritisation process, including estimates of impact and economic efficiency. They often produce these outcomes using a what-if style analysis that explores potential outcomes if a certain allocation is implemented. In some cases, they explore optimising this allocation, e.g., from an economic efficiency perspective. Tools such as LiST and Optima are in this group. Evidence-synthesizing tools combine evidence from different sources to provide a comprehensive picture for their users. By evidence-synthesizing tools, we mean tools that mainly incorporate data that was generated externally and do not further manipulate this data. The HIPtool would fall in this category. Evidence-synthesizing tools inform the discussion in a prioritisation process, and provided the data they synthesize is comparable, they can optimize along the dimension of that data (e.g., find the allocation that is most cost-efficient).[13] Lastly, process tools such as the MCDA tools—PriorityVax and CAPACITI—support the process of prioritisation more broadly. They can help capture and balance values which cannot be readily modelled.

For peer review only - http://bmjopen.bmj.com/site/about/guidelines.xhtml

Which tools should be used depends on the needs of a specific prioritisation exercise, and the tools can often be complementary rather than competitive. For example, MCDA process tools complement tools which have a more technically developed modelling base for modelling disease dynamics or programme costing (the HIPtool can similarly be used in this manner). Indeed, technical tool outputs can be used as inputs to MCDA tools. MCDA-based tools have the potential to bridge the gap between technical analysis and political decision making by highlighting trade-offs between dimensions of value that can be modelled and those which are more difficult to model (e.g., concerns related to justice or economic spillovers). A welcome development would be the conduct of pilot studies that use both technical modelling tools in tandem with MCDA tools to better understand these synergies.

Technical capacity and time available also influence tool choice. Evidence-synthesis tools that do not generate new data may be the simplest to use, particularly if they include pre-populated data. However, this comes at a cost, as their data may come from settings that are different from the context of the given prioritisation exercise. Evidence-generating tools typically require a higher level of technical expertise. Tool design that restricts users' inputs and guides them through the process of conducting an evidence-generating analysis step-by-step simplifies the job for analysts. These restrictions also come at the cost of flexibility, e.g., to contextualise prioritisation exercises. Reducing the amount of data inputs required in these tools also simplifies the process for users, with a similar cost. While MCDA tools may be relatively simple to navigate, MCDA usually requires experienced facilitators alongside the tools to incorporate the views of relevant stakeholders.

No matter the choice of tools, analyses should adhere to good principles of prioritisation, and the tools must provide the opportunity to do so. In our framework, we utilise the iDSI

For peer review only - http://bmjopen.bmj.com/site/about/guidelines.xhtml

Reference Case, which focuses on economic evaluations, to explore tools' adherence to good principles.[1] Though prioritisation involves more than economic evaluation, many of the principles are relevant. In our evaluation, we found that some of the tools do not currently allow users the opportunity to adhere to all principles. For example, a number of tools have limits on the time horizon that may reduce consideration of relevant costs and benefits. Though we focus on the iDSI Reference Case, tools that generate evidence on impact should follow modelling principles in their respective areas (e.g., the disease areas or type of modelling methodology).

The principle of transparency is in some cases violated when tools pre-populate inputs to simplify the process for users but do not provide information about the sources of this data and their underlying assumptions. Pre-populating tools with data and asking users to edit inputs only where needed (to contextualise inputs) is quite attractive; it significantly simplifies the process of implementing an analysis. However, the data should be transparent in its sources and assumptions. Though many tools explicitly state data sources, not all do so. In some cases, they broadly state sources but do not specify them for specific inputs. Pre-populated data may have underlying assumptions, and these are often not clear to tool users. This lack of transparency can reduce trust by the user and may lead to analyses that combine data in a manner that does not necessarily make sense. Tools that do provide pre-populated data offer the option to change this input, potentially mitigating any lack of transparency. However, the mere existence of the pre-populated data may steer users in one direction.

To avoid inappropriate analyses, tools should provide clear documentation on "how" they work in addition to how to use them**.** This recommendation similarly falls under the principle of transparency. Most, but not all, tools already provide sufficient documentation guiding

For peer review only - http://bmjopen.bmj.com/site/about/guidelines.xhtml

users and publications explaining the methodological foundations of models. In some cases, guidance documentation can be simplified and made more accessible; though, we note that the complexity of documentation naturally increases as the complexity of the tool increases (e.g., OneHealth). Explanation of the tool's methodological foundation in peer-reviewed publications can help experts in countries gain trust for the tools. This trust can then be conveyed to other stakeholders. Repositories of the analyses using the tools may also be helpful. Documentation that describes the tool's ownership and process of development and validation can further build trust.

We identified a number of areas where tools can innovate and incorporate outputs that are important for both donors and policymakers. For example, the tools we considered did not make it easy for stakeholders to assess equity. A simple option is to allow disaggregating outputs by subpopulation, and a number of tools already do this; however, tools that do allow disaggregation do not always allow doing this along all dimensions that are important to stakeholders (e.g., wealth). Disaggregating outputs would align with the Disease Control Priorities approach of Extended Cost-Effectiveness Analysis panels.[14] Alternatively, analysts can combine outputs from several runs of a tool using data specific to a given subpopulation each time. However, this puts the onus on tool users (the analysts). Tools could also take novel approaches and help users develop analyses based on distributional cost-effectiveness analysis framework, though data limitations may make this difficult.[15]

An opportunity also exists for building on tools to develop analyses that can compare interventions for different diseases with health system strengthening ones. The OneHealth tool is the most comprehensive among the tools; it incorporates linked modules—which are prioritisation tools themselves focused on specific elements—to be able to make comparisons

For peer review only - http://bmjopen.bmj.com/site/about/guidelines.xhtml

across diseases and investments to strengthen health systems. That complexity comes at a cost: increased resources from users as well as increased difficulty to validate the consistency of the linked modules, which have been combined over the past two decades and were originally developed by different groups. EQUIST, which includes neonatal, infant, and maternal interventions, takes a completely different approach to health system strengthening. It helps identify bottlenecks and estimates the effect of reducing these bottlenecks on effective coverage. It links to other tools such as LiST and OneHealth to then conduct impact and costing analyses.

Our study is limited as it involved only desk-based research that included extracting information from guidance documents and peer-reviewed publications and exploring rudimentary analyses using the tools. Further discussion with tool developers and experienced users would provide a more complete understanding of the tools. However, this limitation can also be viewed as an advantage, as it allowed us to understand the tools based on what is available in the public domain. We held a workshop with tool developers and users, but further interviews with them and stakeholders of prioritisation would provide further insight into the challenges of tool development and their future direction.

Overall, we are encouraged by the plethora of different types of tools available for prioritising resource allocation. There is no one tool that is optimal in every case, and different categories of tools are helpful for different parts of the prioritisation process and for different audiences. As outlined in this discussion, we do see gaps and areas in which the tools included in our analysis can be improved.

For peer review only - http://bmjopen.bmj.com/site/about/guidelines.xhtml

Acknowledgements: We would like to thank participants of the "Why Good Priority-Setting Tools are More Important for Global Health than Ever Before" workshop, which was held in September 2021. The workshop helped refine the research.

Author contributions:
Concept & design: Megiddo, Ruiz, and Morton
Acquisition of data: Blair and Sabei
Analysis & interpretation: Megiddo, Blair, Sabei, and Morton
Drafting of the manuscript: Megiddo, Blair & Sabei
Critical revision of paper for important intellectual content: Megiddo, Blai, Sabei, Ruiz, and Morton
Provision of study materials: Sabei
Obtaining funding: Megiddo, Ruiz and Morton
Administrative, technical, or logistic support: Ruiz
Supervision: Megiddo and Morton

Competing Interests: All authors received support for this study from Bill & Melinda Gates Foundation through the International Decision Support Initiative. Prof Morton and Dr Megiddo reported receiving grants from National Institute of Health Research/ Department of Health and Care Policy Research programme. Prof Morton additionally reported receiving grants from NIHR Global Health Group programme, Chief Scientist's Office Scottish NHS, European Commission, Cancer Research UK, and Pancreatic cancer UK. Prof Morton reported receiving book royalties from Springer. Dr Megiddo additional reported receiving grants from the Scottish Funding Council. Prof Morton reported receiving consulting fees from the Bill & Melinda Gates Foundation via the Thailand Ministry of Public Health and from the WHO Global Malaria Programme. Dr Megiddo reported receiving consulting fees from the International Decision Support Initiative. Prof Morton reported receiving payment or honoraria for lectures from the National University of Singapore. Prof Morton reported participation in the Office of Health Economics board.

Funding: This work received funding support from the Bill and Melinda Gates Foundation (OPP1202541) through the International Decision Support Initiative (iDSI). The funders did not have a role in writing the manuscript or the decision to submit for publication.

Data sharing statement: Data used in the analysis are all publicly available. The Supplementary Materials lists and links to the documentation used in the analysis.

Ethics statements:

Patient consent for publication: not applicable

Ethical approval: not applicable

For peer review only - http://bmjopen.bmj.com/site/about/guidelines.xhtml

References

1. Wilkinson T, Sculpher MJ, Claxton K, et al. The International Decision Support Initiative Reference Case for Economic Evaluation: An Aid to Thought. *Value in Health*. 2016;19(8):921-928. doi:10.1016/j.jval.2016.04.015

2. Glassman A, Chalkidou K, Giedion U, et al. Priority-Setting Institutions in Health: Recommendations from a Center for Global Development Working Group. *Global Heart*. 2012;7(1):13-34. doi:10.1016/j.gheart.2012.01.007

3. Airoldi M, Morton A, Smith JAE, Bevan G. STAR—People-Powered Prioritization: A 21st-Century Solution to Allocation Headaches. *Med Decis Making*. 2014;34(8):965-975. doi:10.1177/0272989X14546376

4. Mitton C, Donaldson C, Dean S, West B. Program budgeting and marginal analysis: a priority-setting framework for Canadian Regional Health Authorities. *Healthc Manage Forum*. 2000;13(4):24-31. doi:10.1016/S0840-4704(10)60790-9

5. Daniels N, Sabin JE. Accountability for reasonableness: an update. *BMJ*. 2008;337:a1850. doi:10.1136/bmj.a1850

6. Baltussen R, Jansen MP, Mikkelsen E, et al. Priority Setting for Universal Health Coverage: We Need Evidence-Informed Deliberative Processes, Not Just More Evidence on Cost-Effectiveness. *Int J Health Policy Manag*. 2016;5(11):615-618. doi:10.15171/ijhpm.2016.83

7. The Global Fund to Fight AIDS, Tuberculosis and Malaria. *Funding Request Instructions*. The Global Fund to Fight AIDS, Tuberculosis and Malaria; 2020. Accessed September 20, 2021. https://www.theglobalfund.org/media/5743/fundingrequest_fullreview_instructions_en.pdf

8. Delva W, Wilson DP, Abu-Raddad L, et al. HIV Treatment as Prevention: Principles of Good HIV Epidemiology Modelling for Public Health Decision-Making in All Modes of Prevention and Evaluation. *PLoS Med*. 2012;9(7). doi:10.1371/journal.pmed.1001239

9. Clapham H, Gad M, Gheorghe A, et al. Assessing fitness-for-purpose and comparing the suitability of COVID-19 multi-country models for local contexts and users. *Gates Open Res*. 2021;5:79. doi:10.12688/gatesopenres.13224.1

10. Policy Group, Covid-19 Multi-Model Collaboration (CMCC). *Guidance on Use of Modelling for Policy Responses to COVID-19*. CMCC; 2020. Accessed August 26, 2020. https://decidehealth.world/sites/default/files/2020-08/CMCC%20Policy%20Group%20Report%20August%202020.pdf

11. McCormick BJJ, Waiswa P, Nalwadda C, Sewankambo NK, Knobler SL. SMART Vaccines 2.0 decision-support platform: a tool to facilitate and promote priority setting for sustainable vaccination in resource-limited settings. *BMJ Global Health*. 2020;5(11):e003587. doi:10.1136/bmjgh-2020-003587

12. Optima HIV User Guide Vol. VI: Parameter Data Sources. Google Docs. Accessed September 6, 2022.

For peer review only - http://bmjopen.bmj.com/site/about/guidelines.xhtml

https://docs.google.com/document/d/1VIxB08GjnLhUjRwLAKuBJ-To2WXud7krK9CNNu6NwIg/edit?usp=drive_open&ouid=106214940111356882136&usp=embed_facebook

13. Fraser-Hurt N, Hou X, Wilkinson T, et al. Using allocative efficiency analysis to inform health benefits package design for progressing towards Universal Health Coverage: Proof-of-concept studies in countries seeking decision support. *PLOS ONE*. 2021;16(11):e0260247. doi:10.1371/journal.pone.0260247

14. Jamison DT, Gelband H, Horton S, et al., eds. *Disease Control Priorities: Improving Health and Reducing Poverty*. Third edition. World Bank Group; 2018.

15. Cookson R, Griffin S, Norheim OF, Culyer AJ, eds. *Distributional Cost-Effectiveness Analysis: Quantifying Health Equity Impacts and Trade-Offs*. Oxford University Press; 2020. Accessed September 23, 2021. https://oxfordmedicine.com/view/10.1093/med/9780198838197.001.0001/med-9780198838197

For peer review only

Supplementary Material 1. Tool documentation

CAPACITI (Country-led Assessment for Prioritization in Immunization)
Guidance manual

PriorityVax
User guide
Phase 1 report
Phase 2 report
Phase 3 report

Optima
HIV User Guide
TB User Guide
HIV allocative efficiency analysis training workshop agenda and training materials
TB allocative efficiency analysis training workshop agenda and training materials
Software Reference Manual
Expenditure Data Guide
HIV-related Abbreviations
Parameter Data Sources

OneHealth
FAQ
Startup Manual
Technical Notes
Technical Notes Annexes
Intervention Input Assumptions Manual
Defining the target population PIN and coverage in OneHealth
Using OneHealth for programme planning
MNS Module Manual
Manual

LiST (Lives Saved Tool)
Manual
Subnational modeling
Nutrition modeling in LiST

EQUIST (Equitable strategies to save lives)
Technical notes
User guide

For peer review only

Supplementary materials 2. Tables

Table S2.1. Tools additional information

	Year created	Owned / developed by	Time/process to implement	Required level of technical expertise
CAPACITI	2020	WHO	A typical recommendation is expected to take from 4 to 6 months. Intensive data collection required.<br><br>1. Clarify decision question<br>2. Set criteria for decision-making<br>3. Collect and assess evidence<br>4. Appraisal of results<br>5. Finalisation and communication of recommendation	Minimal (No/minimal training is required)
PriorityVax	2010**	Sabin Vaccine Institute	No information given on timing of process.<br><br>1. Policy questions are defined (Define decision criteria, Discuss and identify attributes, Explicitly weight criteria, Discuss and weight attributes)<br>2. PICO questions are defined (Population, Intervention, Comparator, Outcome)<br>3. Evidence gathered and reviewed to address PICO questions and evidence attributes<br>4. Evidence used to extract values and/or score attributes<br>5. Recommendation provided<br>6. Interactive sensitivity analysis	Minimal (No/minimal training is required)
HIPtool	NA	WB	No formal information on process, intensive data collection required.	Minimal (No/minimal training is required)
Optima	2004	OPTIMA consortium	No formal information on process, intensive data collection required.	Detailed knowledge required (3 days intensive training)
OneHealth (Spectrum suite)	1997	Avenir Health	A typical recommendation is expected to take from 4 to 6 months:<br><br>1. Initiation of planning process.<br>2. Stakeholder meeting (first plenary).<br>3. Tool set-up with baseline data entry.<br>4. Second plenary.<br>5. Individual modules/groups.<br>6. Assessing the joint plan.<br>7. Adjusting individual modules/groups.<br>8. Submission of revised modules.<br>9. Group discussion and final presentation of NHSP (National health service plan)	Detailed knowledge required
LiST	2003	Institute for International Programs at Johns Hopkins Bloomberg School of Public Health	No information in user manual or technical notes on likely process and timelines for implementation	Minimal (No/minimal training is required)
EQUIST	2016	UNICEF in association with Johns Hopkins Bloomberg School of Public Health and WB	No information given on timing of process.<br><br>Users conduct a situational analysis, comprising an equity profile analysis and an equity frontier analysis, followed by a scenario analysis, as follows:<br><br>1. Define priority / target populations<br>2. Prioritise epidemiological priorities<br>3. Prioritise interventions<br>4. Prioritise key bottlenecks	Minimal (No/minimal training is required)

For peer review only

			5. Prioritise causes of bottlenecks<br>6. Select strategies to address cause of bottleneck<br>7. Assess expected impact and cost effectiveness	

CAPACITI: Country-led Assessment for Priortization in Immunization; HIPtool: Health Interventions Priortization tool; LiST: Lives Saved Tool; EQUIST: Equitable strategies to save lives; LMIC: Low- and Middle-Income Country; MNCH&N: Maternal, Newborn, Child Health and Nutrition.

* Adaptability to extend to new diseases and interventions.

** The Institute of Medicine commissioned the tool in 2010, and it was developed with the name SmartVaccines. This evolved into PriorityVax by 2018.

For peer review only

BMJ Open

An evaluation framework study assessing the role, applicability, and adherence to good practice of planning support tools for allocation of development aid for health in low- and middle-income countries

Journal:	*BMJ Open*
Manuscript ID	bmjopen-2022-069590.R1
Article Type:	Original research
Date Submitted by the Author:	26-Apr-2023
Complete List of Authors:	Megiddo, Itamar; University of Strathclyde, Department of Management Science<br>Blair, Shona; University of Strathclyde, Department of Management Science<br>Sabei, Davood; University of Strathclyde, Department of Management Science<br>Ruiz, Francis; London School of Hygiene & Tropical Medicine, Department of Global Health and Development; Center for Global Development<br>Morton, Alexander; University of Strathclyde
**Primary Subject Heading**:	Global health
Secondary Subject Heading:	Health economics, Health policy
Keywords:	Health economics < HEALTH SERVICES ADMINISTRATION & MANAGEMENT, Health policy < HEALTH SERVICES ADMINISTRATION & MANAGEMENT, International health services < HEALTH SERVICES ADMINISTRATION & MANAGEMENT, HEALTH ECONOMICS

SCHOLARONE™
Manuscripts

I, the Submitting Author has the right to grant and does grant on behalf of all authors of the Work (as defined in the below author licence), an exclusive licence and/or a non-exclusive licence for contributions from authors who are: i) UK Crown employees; ii) where BMJ has agreed a CC-BY licence shall apply, and/or iii) in accordance with the terms applicable for US Federal Government officers or employees acting as part of their official duties; on a worldwide, perpetual, irrevocable, royalty-free basis to BMJ Publishing Group Ltd ("BMJ") its licensees and where the relevant Journal is co-owned by BMJ to the co-owners of the Journal, to publish the Work in this journal and any other BMJ products and to exploit all rights, as set out in our licence.

The Submitting Author accepts and understands that any supply made under these terms is made by BMJ to the Submitting Author unless you are acting as an employee on behalf of your employer or a postgraduate student of an affiliated institution which is paying any applicable article publishing charge ("APC") for Open Access articles. Where the Submitting Author wishes to make the Work available on an Open Access basis (and intends to pay the relevant APC), the terms of reuse of such Open Access shall be governed by a Creative Commons licence – details of these licences and which Creative Commons licence will apply to this Work are set out in our licence referred to above.

Other than as permitted in any relevant BMJ Author's Self Archiving Policies, I confirm this Work has not been accepted for publication elsewhere, is not being considered for publication elsewhere and does not duplicate material already published. I confirm all authors consent to publication of this Work and authorise the granting of this licence.

For peer review only

An evaluation framework study assessing the role, applicability, and adherence to good practice of planning support tools for allocation of development aid for health in low- and middle-income countries

Itamar Megiddo, PhD[1,*], Shona Blair, PhD[1], Davood Sabei, PhD[1], Francis Ruiz, MSc[2,3], Alec Morton, PhD[1]

[1] Department of Management Science, University of Strathclyde, Glasgow, UK
[2] Department of Global Health and Development, London School of Hygiene and Tropical Medicine, London, UK.
[3] Center for Global Development Europe, London, UK.

Dr Itamar Megiddo
Department of Management Science
University of Strathclyde
199 Cathedral Street
Glasgow, G4 0QU
Email: itamar.megiddo@strath.ac.uk
Phone: 44 (0)141 548 3452

Key words: development aid for health (DAH); health planning tools; prioritization.

For peer review only

Abstract

Objectives: Allocation of Development Aid for Health (DAH) is controversial and challenging. In recent years, several planning-software tools have promised to help decision-makers align resource allocation with their objectives, more clearly connect prioritisation to evidence and local circumstances, and increase transparency and comparability. We aim to explore these tools to provide insight into their fitness for purpose and suggest future directions to fulfil that promise.

Design: We identified seven tools that met the inclusion criteria and developed an evaluation framework to compare them along two dimensions for assessing fitness for pupose: ability to produce analyses adhering to principles laid out in the International Decisions Support Initiative (iDSI) Reference Case for health economic evaluations; and resources required, including expertise and time. We extracted information from documentation and tool use and sent this information to tool developers for confirmation.

Results: We categorise the tools into evidence-generating ones, evidence-syntheses ones, and process support ones. Tools' fitness for purpose varies by the context, technical capacity, and time limitation. The tools adhere to several Reference Case principles but often not to all of them. The source and underlying assumptions of pre-populated data are often opaque. Comparing vertical interventions across diseases and health system strengthening ones remains challenging.

Conclusions: The plethora of tools that aid priority setting in different ways is encouraging. Developers and users should place further emphasis on their ability to produce analyses that

For peer review only

adhere to prioritisation principles. Opportunities for further development include using evidence-generating tools and Multi-Criteria Decision Analysis approaches complimentarily. However, maintaining tool simplicity should also be considered to allow wider access.

For peer review only

Strengths and limitations of the study

- The study presents a framework for objectively evaluating tools for national health planning, while noting that context matters.
- The tables provided in the study allow for quick comparison of assumptions, features, and required resources across the tools, providing information on their fitness for purpose in a given context.
- The study was limited to desk-based research, which included extracting information from guidance document and peer-reviewed publications, as well asconducting rudimentary analyses using the tools.
- By focusing on publicly available information, the study team avoided having more information than country-level analysts or other stakeholders who use the tools' outputs.
- In-depth interviews with stakeholders of prioritization could provide additional insight into the challenges of tool development and their future direction.

For peer review only - http://bmjopen.bmj.com/site/about/guidelines.xhtml

Introduction

National health planning tools that simplify the process of priority setting have the potential to aid in adhering to the guiding principles of prioritisation. Allocation of development aid for health (DAH) is always controversial and challenging, and the emergence of COVID-19 as a fourth major internationally endemic communicable disease exacerbates the difficulty of overcoming these challenges in a more constrained financial environment. The guiding principles suggest that prioritisation should transparently reflect the values of the country—represented through stakeholders—and this requires a shared understanding across stakeholders of the prioritisation process and the evidence used in this process.[1] Gaining this shared understanding may be difficult due to the differing knowledge base among stakeholders on health interventions and technical methods for evaluating them. In recent years, research groups and global health and development organisations have developed tools for national health planning in low- and middle-income countries (LMICs). These tools simplify comparison across programmes and can inform the discussion in the prioritisation process and help develop an evidence base that supports decision-makers at the country level.

It is important to realise that tools support rather than make decisions. A key insight from Health Technology Assessment experience in high-income countries is that effective priority setting requires both technical methods and tools on the one hand and institutions and institutional processes on the other.[2] A number of different process frameworks have been advocated for prioritisation decisions: Socio-Technical Allocation of Resources (STAR),[3] Program Budgeting and Marginal Analysis,[4] Accountability for Reasonableness,[5] and Evidence-Informed Deliberative Process.[6] Though tools and the processes they support are often difficult to separate, in this paper we are agnostic about how the prioritisation process should be structured. We define a *national health planning tool* as a software package that

For peer review only - http://bmjopen.bmj.com/site/about/guidelines.xhtml

requires input and produces outputs specifically designed to support country-level decision-makers in planning. In the rest of the paper, we restrict our analysis and discussion to tools that provide a quantitative analysis, but their purpose may be to generate evidence for use as part of the prioritisation process or to support the process in other ways. Our focus is on these tools' applicability and ability to support or produce analyses that are useable within the context of some structured prioritisation process.

Tool developers make several choices that influence the tools' applicability in these contexts, including ones affecting the required level of expertise to use them and users' ability to adhere to good practice. Tools can offer users more or less flexibility, though they all limit this flexibility to some extent. Unlimited flexibility does away with the tool and leaves all decisions up to the analysts conducting the prioritisation exercise. This flexibility has benefits such as improving the analysts' ability to utilise the most novel methods and not restricting contextualisation (beyond data availability limitations). However, it also reduces access to conducting prioritisation to those with very high levels of expertise and increases the time costs of a given exercise. The choices made by tool developers limit that flexibility while defining the inherent 'tool opinion' about what ingredients are important for its given purpose. Choices that restrict specific inputs (e.g., the number of years into the future considered) may limit users' ability to adhere to certain prioritisation guiding principles, while in other cases, they can guide or steer—and in some cases force—users to adhere to certain principles, exhibiting that 'tool opinion'.

Further, the choices made on the presentation of tools' outputs affect accessibility for users of these outputs. The sheer range of stakeholders that contribute to priority setting can lead to demands for different types of outputs. Their differing knowledge base can contribute to

For peer review only - http://bmjopen.bmj.com/site/about/guidelines.xhtml

information asymmetries in the prioritisation process and the evidence it uses. The Global Fund to Fight Aids, Tuberculosis, and Malaria (GFATM), for example, utilises country coordinating mechanisms (CCMs) to submit applications and govern its DAH funds. These CCMs are responsible for empowering and representing the views of stakeholders from across sectors (e.g., academic institutions, civil society, government, those with lived experience, NGOs, and private institutions), and they are asked to submit an application for funding that includes a description of their prioritisation process.[7] A tool with output that simplifies the understanding of evidence or improves the process to incorporate the disparate voices would go a long way toward achieving the vision for CCMs. However, asking tools to cater to all of these stakeholders as well as to donor agendas—that often require information on impact, value for money, and equity—is a tall order.

The global health community has a limited grasp on the success of these tools' fulfilment of the promise to increase access to conducting and utilising the outputs of prioritisation exercises, in a way that is consistent with the principles of good prioritisation. This gap makes it difficult to decide on the context in which one tool should be used rather than another. The World Bank has explored tools for allocative efficiency within the context of HIV, explaining the purpose of a few tools and broadly discussing some of their limitations. The literature on good principles for infectious disease and health economic modelling in LMICs and communication of these models is more mature.[1,8] A recent study, compared COVID-19 models along dimensions of model characteristics and reporting.[9,10] Based on these elements, the study defined a framework for thinking about the models' fitness for purpose, setting questions to guide both output users—policymakers in this case—and analysts that adapt these models to contextualise an analysis.[9] Both of these audiences are also important for characterising *national health planning tools* that support priority setting.

For peer review only - http://bmjopen.bmj.com/site/about/guidelines.xhtml

Our paper aims to provide an understanding of these tools' characteristics that influence their suitability for specific purposes and to gain insights into their future directions of development and use. We develop an evaluation framework that considers the tools' ability to produce analyses or processes that adhere to good practice and the onus they put on their users in terms of expertise and time. We explore the availability of transparent documentation on the tools and the ease of navigating this documentation. We further describe the types of outputs they produce. We *do not* rank the tools or suggest that any are inadequate for some part of the priority setting process. In the following sections, we describe our methodology for developing and utilising our evaluation framework, describe our findings, and discuss conclusions and recommendations.

Methods

Tool selection

We selected planning software tools that aid country-level decision-makers in resource allocation based on the following inclusion criteria: the software package i) provides a quantitative analysis, ii) is applicable for multiple countries, iii) can be adapted to utilise new data, and iv) is available in the public domain during the evaluation period.

In our search strategy, we sought to identify relevant tools by leveraging the knowledge of our project team and partners and surveying them as well as conducting targeted searches of country investment cases. We followed the search by filtering the tools through the inclusion criteria. Since there are no standard terms that can be used to describe these software tools as a class, we searched for tools that have been previously employed in resource allocation decisions or have received recent investments and backing from global health organisations, indicating their potential future use. The cut-off data for tools we considered was January

For peer review only - http://bmjopen.bmj.com/site/about/guidelines.xhtml

2021. Our partners have been active in the field for many years and possess good knowledge of the landscape. Nonetheless, we augmented this knowledge with the targeted searches to not overlook relevant tools.

During the initial step, we conducted discussions with our partners and identified several pertinent tools, including the OneHealth Tool in combination with the Spectrum suite, Optima, Health Interventions Prioritisation (HIP) tool, PriorityVax, Country-led Assessment for Prioritisation on Immunization (CAPACITI) decision-support tool, STAR, and Cascade. In the subsequent step, in targeted searches, we and project partners at the Centre for Global Development reviewed funding requests and investment cases submitted to GFATM and Global Financing Facility, a multi-stakeholder partnership hosted by the World Bank.[7,11,12] This search confirmed the use of several tools we had identified in the initial step and additionally identified EQUitable Strategies to Save Lives Tool (EQUIST).

Finally, we identified seven tools that met our criteria among the tools we found in our search. LiST is part of the Spectrum suite, but we include it separately as it has been used standalone for many analyses. We excluded two tools we found in our initial search as they did not meet inclusion criteria (i) and (iv); STAR and Cascade respectively. STAR is a process framework for allocation and does not include a tool that provides quantitative outputs. Cascade is an online tool, and its website was down when the analysis was conducted.

Development of evaluation framework

The evaluation framework is based on good practices of economic analyses as outlined by the iDSI Reference Case[1] as well as on the requirements for utilising the tools. The reference

For peer review only - http://bmjopen.bmj.com/site/about/guidelines.xhtml

case lists eleven principles for economic evaluations (Table 1), and these were adapted as relevant for the resource allocation tools and organized in a set of four topics (with corresponding tables): modelling assumptions, model inputs, model outputs, and validity and transparency. Two additional topics (and corresponding tables) that do not correspond to the reference case principles provide an overview of the tools and describe the resources—in time and expertise—required for using the tools and understanding their outputs. Table 1 describes the corresponding principles for each evaluation table.

Table 1. Evaluation framework and the iDSI Reference Case Principles

Evaluation Framework Table	Principles
Tool overview and purpose	Does not correspond to principles
Modelling assumptions adherence to reference case	Principle 6 – Time horizon and discounting
	Principle 7 – The analysis perspective (non-health effects and costs outside the health budget should be included)
Resources and data requirements	Resource requirements do not correspond to principles
	Principle 3 – Systematic and comprehensive evidence gathering
	Principle 5 – Costs reflect all resources used
	Principle 8 – Heterogeneity in population subgroups should be explored
Outputs	Principle 4 – Appropriate measures of outcome
	Principle 8 – Heterogeneity in population subgroups should be explored
	Principle 9 – Uncertainty
	Principle 10 – Impact on budget and other constraints identified
	Principle 11 – Explore equity
Transparency and validity	Principle 1 – Transparency

We examined whether the tools allow users to adhere to these principles, as violating them in a particular prioritisation exercise will also depend on how the tools are used. We dropped Principle 2—the comparator accurately reflects the decision problem—which was

For peer review only - http://bmjopen.bmj.com/site/about/guidelines.xhtml

particularly difficult to capture without considering the tool users' decisions. However, we discuss the limitations to tools' adaptability that may prevent extending analyses to consider additional diseases and interventions (Tools overview and purpose). This discussion should provide sufficient information on the limitations to comparators the user can evaluate.

Each table consists of items used in the data extraction process. Items do not evaluate appropriateness or adherence to the principles. Rather, they report information about the tools characteristics that can be used to evaluate adherence to these principles as well as the level of knowledge needed to use them.

Data extraction

Two members of the research team extracted information for the seven tools we evaluated on the items identified for each table in the evaluation framework. They experimented with the tools and extracted information based on documentation and publications on the tool (see documentation in Supplementary Materials 1). To assess usability, they explored tool documentation and, where available, identified the level of user expertise developers assumed or suggested. They then developed and implemented rudimentary analyses using the tools' interfaces to understand how quickly they could set up an analysis after reading the documentation. After this extraction from documents and experiments with the tools, the entire research team then discussed the data in the evaluation tables to develop the analysis.

Patient and public involvement

Patients and the public were not involved in this study.

For peer review only - http://bmjopen.bmj.com/site/about/guidelines.xhtml

Results

Tools overview and purpose

The tools can be categorised along different dimensions that help identify their role in a prioritisation exercise, including by their aims and diseases and interventions they include in an analysis (Table 2). Apart from EQUIST and Optima, the tools are not limited by diseases they can analyse.

Table 2. Tools overview and purpose

	Stated aim(s) of tool	Approach & theoretical foundations	Adaptability to extend (e.g., custom interventions)
CAPACITI	Support evaluation of immunisation options incorporating values and programme context	MCDA embedded in broader problem-structuring framework	Focus on immunisation programmes but can extend to other
PriorityVax	Support vaccine prioritisation; clarify data uncertainty impact; facilitate discussion; clarify stakeholder views	MCDA embedded in broader problem-structuring framework	Focus on immunisation programmes but can extend to toher
HIPtool	Support health intervention prioritisation at country level	Evidence-synthesis	Yes
Optima	Analyse and project HIV and TB epidemics; determine optimal resource allocations	Impact model: dynamic compartmental models; Costing: logistic cost function model; Global optimization to minimize outcome (e.g., DALYs) or costs to achieve defined outcomes	No
OneHealth (Spectrum suite)	Support to inform national health care planning and resource needs at country level, strengthening health system analysis, costing, and finance	Impact model: module specific (e.g., LiST for child and maternal health interventions, or TIME a dynamic compartmental model for TB); Costing: ingredients-based costing	Custom interventions can be added

For peer review only - http://bmjopen.bmj.com/site/about/guidelines.xhtml

LiST	Estimate the impact of scaling up MNCH&N interventions in LMICs	Impact model: static equation-based model; costing: ingredient-based costing	Focus on MNCH&N; Custom interventions can be added; only LMICs
EQUIST	Estimate the impact of scaling up MNCH&N interventions in LMICs	Bottleneck analysis and statistical analysis of changes to effective coverage; Impact analysis: based on LiST; Costing: based on LiST and OneHealth	Focus on MNCH&N; only LMICs

CAPACITI: Country-led Assessment for Priortization in Immunization; HIPtool: Health Interventions Priortization tool; LiST: Lives Saved Tool; EQUIST: Equitable strategies to save lives; LMIC: Low- and Middle-Income Country; MNCH&N: Maternal, Newborn, Child Health and Nutrition.
* Adaptability to extend to new diseases and interventions.

For peer review only

Further exploring the approaches and methods the tools use, we can categorize them into ones that generate new data (e.g., through impact and costing models), ones that synthesize existing data, and ones that focus on problem structuring and process (Table 2). Tools that generate new data produce predictions of intervention impact using simulation models. The HIPtool synthesizes evidence, relying on evidence generated in external studies. Process tools provide a broader problem-structuring framework such as multi-criteria decision analysis (MCDA; e.g., PriorityVax). They similarly rely on evidence generated in external studies, but their focus is on the process of the decision-making exercise. Different elements of EQUIST lie in different categories: the tool synthesises evidence to display outputs on bottlenecks and intervention prioritisation; and it guides users on process in a seven-step approach that includes stakeholder discussion.

Modelling assumptions adherence to reference case

The flexibility the tools provide users (e.g., to conduct analyses from different perspectives), their built-in assumptions, and the directions they steer users have implications for users' ability to develop analyses that adhere to the iDSI Reference Case principles (Table 3). The principles suggest analyses report a disaggregated societal perspective. Several tools allow

For peer review only - http://bmjopen.bmj.com/site/about/guidelines.xhtml

users to conduct evaluations from different perspectives. Other tools focus on the health system perspective. A few of the tools restrict the time horizon to 2050, limiting the possibility to consider a lifetime horizon that is recommended by the principles. In evidence-synthesis and process-focused tools, the time horizon depends on the evidence they incorporate and the transparency and accessibility the tool provides users to its sources (if users use pre-populated data). Most tools allow users to conduct parameter sensitivity and uncertainty analyses. They do not consider stochastic uncertainty—random variation in outcomes—or uncertainty in the analysis structure.

Table 3. Modelling Assumptions

	Theoretical basis	Perspective	Time horizon	Forms of uncertainty explicitly considered*
CAPACITI	Multi-criteria decision analysis (MCDA) embedded in broader problem-structuring framework	Flexible	N/A	Parameter; Criteria weighting
PriorityVax	Multi-criteria decision analysis (MCDA) embedded in broader problem-structuring framework	Flexible	N/A	Parameter; Criteria weighting
HIPtool	Synthesise evidence	Health system	N/A	
Optima	Impact model: dynamic compartmental models; costing: logistic cost function model; global optimization to minimize outcome (e.g., DALYs) or costs to achieve defined outcomes	Health system	Do not know	Parameter
OneHealth (Spectrum suite)	Impact model: module specific (e.g., LiST for child and maternal health interventions, or TIME a dynamic compartmental model for TB); costing: ingredients-based costing	Flexible	100 years fixed time horizon	Parameter
LiST	Impact model: static equation-based model; costing: ingredient-based costing	Flexible	Maximum until 2050	Parameter

For peer review only - http://bmjopen.bmj.com/site/about/guidelines.xhtml

EQUIST	Bottleneck analysis and statistical analysis of changes to effective coverage based on strategies' bottleneck reduction; impact analysis: based on LiST; costing: based on LiST and OneHealth	Health System	Maximum until 2050	

CAPACITI: Country-led Assessment for Priortization in Immunization; HIPtool: Health Interventions Priortization tool; LiST: Lives Saved Tool; EQUIST: Equitable strategies to save lives.
* The two forms of uncertainty in the table include: (i) Parameter – uncertainty in tool input values; (ii) Criteria weighting – uncertainty in the relative importance of criteria (MCDA specific). Users can explore different scenarios using the tools, including EQUIST, which can be used to assess uncertainty. As this feature is not automated but requires developing new analyses we do not include it in the table. Other forms of uncertainty such as model structural uncertainty are not explicitly considered by the tool and are thus not included.

Resource and data requirement

The required technical expertise and time investment vary with tool complexity (Table 1 in Supplementary Materials 2). Relatively novice users can implement analyses in tools that merely synthesise information. Though, the users do require an understanding of health economic evaluation terminology. Users need knowledge across a range of modules for more complex tools such as OneHealth and Optima. The tools do not consistently provide guidelines on the time an analysis requires. While tools such as CAPACITI and OneHealth recommend an analysis over four to six months, HIPtool does not require significant time investment if analysts and stakeholders use its pre-populated data.

Most tools require intensive data collection to conduct a contextualised analysis, albeit most, incorporate pre-populated data (Table 4). Where they do not provide pre-populated data, they provide guidance on data collection and discuss sources in their literature.[13] The level of guidance varies by tool. Many of the tools reference commonly known global health data sources (e.g., the World Health Organization and the Global Burden of Disease [GBD] study). Additionally, they reference specific studies (e.g., OPTIMA).[14] We observed differing transparency levels about tools' data sources. PriorityVax, for example, provided a thorough discussion of data sources to support analysis. HIPtool noted its use of the GBD, Disease Control Priorities (DCP3) Essential Universal Health Coverage package and Highest-Priority

For peer review only - http://bmjopen.bmj.com/site/about/guidelines.xhtml

Package data and "other secondary sources." The tool and its documentation do not provide the specific source and underlying assumptions of pre-populated data such as incremental cost-effectiveness ratios (ICERs).

Table 4. Data requirements

	Target population	Epidemiological	Intervention coverage	Intervention cost*	Preloaded data
CAPACITI	Flexible	Flexible	NA	Flexible	No
PriorityVax	Flexible	Flexible	NA	Flexible	Upon request
HIPtool	NA	Burden prevalence	Not an input; optimises investment	Intervention unit costs	Yes
Optima	By subpopulation (demographic, geography, and risk-group) and population dynamics**	Several inputs	Over time	Intervention unit costs	Yes (sources in documentation)
OneHealth (Spectrum suite)	By subpopulation (socio-demographic and geography)***; population dynamics **	Several inputs (varies by module)	Over time	Intervention; human-resources, logistics, and infrastructure for delivery	Yes (sources in tool)
LiST	By subpopulation (socio-demographic and geography); population dynamics**	Incidence, prevalence, and health indicators, depending on disease	Over time	Intervention; human-resources, logistics, and infrastructure for delivery	Yes (sources in tool)

For peer review only - http://bmjopen.bmj.com/site/about/guidelines.xhtml

EQUIST	By subpopulations (socio-demographic and geography)	Several inputs	Bottlenecks to effective coverage and their contribution	Yes	Yes (sources in tool); several inputs cannot be updated by the user but can be managed by the "Country administrator"

CAPACITI: Country-led Assessment for Priortization in Immunization; HIPtool: Health Interventions Priortization tool; LiST: Lives Saved Tool; EQUIST: Equitable strategies to save lives.
NA – not applicable; several of the categories are not applicable for evidence-synthesis and/or process tools such multi-criteria decision analysis (MCDA) ones; Flexible – as MCDA tools define criteria as part of the process, these inputs are flexible
* Users can combine costs to include them under one category; we note how tools disaggregate the cost data further (e.g., human resources and logistics).
** Population dynamic parameters include ones related to fertility and mortality (exogenous to diseases and interventions evaluated).
** The types of data included may vary by module within OneHealth.

The types of data tools require depends on the tools' aims (Table 4). For example, tools that estimate impact require detailed population and epidemiological data. EQUIST, which incorporates a bottleneck analysis, inputs include information about bottlenecks to effective coverage. The MCDA-based tools data requirements depend on the criteria decision-makers and stakeholders deem important. These tools can also include inputs such as ICERs, which tools that generate new data often consider as outputs. HIPtool, similarly, considers ICERs as an input. The HIPtool also requires equity and financial risk protection scores (and provides pre-populated values).

Outputs

Most tools use visualisation to present outputs on cost, health outcomes (or disease burden), and economic efficiency, with MCDA tools also including aggregated scores (Table 5). Both the HIPtool and Optima outputs include a comparison between baseline and optimal scenarios in terms of economic efficiency.

For peer review only - http://bmjopen.bmj.com/site/about/guidelines.xhtml

Table 5. Outputs

	Cost	Health outcomes	Economic efficiency	Type(s) of output	Uncertainty representation	Outputs can be disaggregated into subpopulations
CAPACITI	Yes	Yes	Yes	Aggregate scores, Charts, Tables	No	No
PriorityVax	Yes	Yes	Yes	Aggregate scores, Charts, Tables	No	Scores based on entire population
HIPtool	Yes	Yes	Yes, including optimal package of interventions	Charts	No	No
Optima	Yes	Yes	Yes, including optimal package of interventions	Charts, Tables	Yes, plots with parameter scenarios	Demographic and geospatial characteristics
OneHealth (Spectrum suite)	Yes	Yes	Yes	Charts, Tables	Yes, uncertainty bounds	Demographic characteristics
LiST	Yes	Yes	Yes	Charts, Tables	Yes, uncertainty bounds	Demographic characteristics
EQUIST	Yes	Yes	Yes	Charts, Tables	No	Demographic charecteristics, wealth, and geography

CAPACITI: Country-led Assessment for Priortization in Immunization; HIPtool: Health Interventions Priortization tool; LiST: Lives Saved Tool; EQUIST: Equitable strategies to save lives.

For peer review only

Only some of the tools present uncertainty in their outputs (Table 5). Uncertainty bounds are the most common representation of uncertainty among these tools. While the MCDA tools outputs do not to explicitly describe uncertainty in results, they recommend users conduct a sensitivity analysis to understand variation due to stakeholder preferences and criteria values uncertainty.

Several, but not all, tools allow users to disaggregate results by different demographic dimensions (Table 5). While MCDA tools do not present disaggregated outputs, they can include subpopulation outcomes as criteria. EQUIST disaggregates some outputs by wealth. The HIPtool does not produce disaggregated outputs.

Transparency and validity

The majority of the tools provided some degree of documentation; however, we found variation in the accessibility of the documentation (Table 6). All tools have produced at least one analysis that was published in a peer reviewed journal. Frequently, such publications were written by the tool developers themselves, although not in all cases. Given the variety of release dates of the tools (Table 1 in the Supplementary Materials), the lack of independent tool usage does not necessarily imply a lack of tool usage by other groups.

Table 6. Transparency and Validity

	Documentation availability*	Limitations explained	Interests of authors declared	Funding declared	Open source	Peer reviewed publications
CAPACITI	Yes	Yes, but no dedicated	No	No	Yes	Yes

		limitations section				
PriorityVax	Yes	Yes, but no dedicated limitations section	No	Yes	No	Yes
HIPtool	No (work in progress)	No	No	No	No	Yes
Optima	Yes	Yes	Yes	Yes	No	Yes
OneHealth (Spectrum suite)	Yes	Yes	Yes	Yes	No	Yes
LiST	Yes	Yes	Yes	Yes	No	Yes
EQUIST	Yes	Yes, but no dedicated limitations section	No	Yes	No	Yes

CAPACITI: Country-led Assessment for Priortization in Immunization; HIPtool: Health Interventions Priortization tool; LiST: Lives Saved Tool; EQUIST: Equitable strategies to save lives.
* This included both guidance manuals and technical reports. Other forms of documentation (presentations, online discussions, secondary reports) were available for some tools; however, these have not been included in the evaluation.

For peer review only

Discussion and conclusion

The promise of health planning tools is that they will simplify the process of prioritising health interventions and resource allocation in a manner that consistently adheres to accepted principles while also creating trust and a shared understanding among stakeholders. In this study, we developed and implemented an evaluation framework to describe tool characteristics that convey where the tools succeed and provide insight into potential future directions for tool developers. Our hope is that comparing tool characteristics also helps tool users identify which tool—or tools—are well suited for their prioritisation exercise.

For peer review only - http://bmjopen.bmj.com/site/about/guidelines.xhtml

Based on our findings on tool purpose and the types of inputs they require, we group the tools into three nonexclusive categories: evidence-generating, evidence-synthesising, and process. Evidence-generating tools produce outputs used in the prioritisation process, including estimates of impact and economic efficiency. They often produce these outcomes using a what-if style analysis that explores potential outcomes if a certain allocation is implemented. In some cases, they explore optimising this allocation, e.g., from an economic efficiency perspective. Tools such as LiST and Optima are in this group. Evidence-synthesizing tools combine evidence from different sources to provide a comprehensive picture for their users. By evidence-synthesizing tools, we mean tools that mainly incorporate data that was generated externally and do not further manipulate this data. The HIPtool would fall in this category. Evidence-synthesizing tools inform the discussion in a prioritisation process, and provided the data they synthesize is comparable, they can optimize along the dimension of that data (e.g., find the allocation that is most cost-efficient).[15] Lastly, process tools such as the MCDA tools—PriorityVax and CAPACITI—support the process of prioritisation more broadly. They can help capture and balance values which cannot be readily modelled.

Which tools should be used depends on the needs of a specific prioritisation exercise, and the tools can often be complementary rather than competitive. For example, MCDA process tools complement tools which have a more technically developed modelling base for modelling disease dynamics or programme costing (the HIPtool can similarly be used in this manner). Indeed, technical tool outputs can be used as inputs to MCDA tools. MCDA-based tools have the potential to bridge the gap between technical analysis and political decision making by highlighting trade-offs between dimensions of value that can be modelled and those which are more difficult to model (e.g., concerns related to justice or economic spillovers). A

For peer review only - http://bmjopen.bmj.com/site/about/guidelines.xhtml

welcome development would be the conduct of pilot studies that use both technical modelling tools in tandem with MCDA tools to better understand these synergies.

Technical capacity and time available also influence tool choice. Evidence-synthesis tools that do not generate new data may be the simplest to use, particularly if they include pre-populated data. However, this comes at a cost, as their data may come from settings that are different from the context of the given prioritisation exercise. Evidence-generating tools typically require a higher level of technical expertise. Tool design that restricts users' inputs and guides them through the process of conducting an evidence-generating analysis step-by-step simplifies the job for analysts. These restrictions also come at the cost of flexibility, e.g., to contextualise prioritisation exercises. Reducing the amount of data inputs required in these tools also simplifies the process for users, with a similar cost. While MCDA tools may be relatively simple to navigate, MCDA usually requires experienced facilitators alongside the tools to incorporate the views of relevant stakeholders.

No matter the choice of tools, analyses should adhere to good principles of prioritisation, and the tools must provide the opportunity to do so. In our framework, we utilise the iDSI Reference Case, which focuses on economic evaluations, to explore tools' adherence to good principles.[1] Though prioritisation involves more than economic evaluation, many of the principles are relevant. In our evaluation, we found that some of the tools do not currently allow users the opportunity to adhere to all principles. For example, a number of tools have limits on the time horizon that may reduce consideration of relevant costs and benefits. Though we focus on the iDSI Reference Case, tools that generate evidence on impact should follow modelling principles in their respective areas (e.g., the disease areas or type of modelling methodology).

For peer review only - http://bmjopen.bmj.com/site/about/guidelines.xhtml

The principle of transparency is in some cases violated when tools pre-populate inputs to simplify the process for users but do not provide information about the sources of this data and their underlying assumptions. Pre-populating tools with data and asking users to edit inputs only where needed (to contextualise inputs) is quite attractive; it significantly simplifies the process of implementing an analysis. However, the data should be transparent in its sources and assumptions. Though many tools explicitly state data sources, not all do so. In some cases, they broadly state sources but do not specify them for specific inputs. Pre-populated data may have underlying assumptions, and these are often not clear to tool users. This lack of transparency can reduce trust by the user and may lead to analyses that combine data in a manner that does not necessarily make sense. Tools that do provide pre-populated data offer the option to change this input, potentially mitigating any lack of transparency. However, the mere existence of the pre-populated data may steer users in one direction.

To avoid inappropriate analyses, tools should provide clear documentation on “how” they work in addition to how to use them**.** This recommendation similarly falls under the principle of transparency. Most, but not all, tools already provide sufficient documentation guiding users and publications explaining the methodological foundations of models. In some cases, guidance documentation can be simplified and made more accessible; though, we note that the complexity of documentation naturally increases as the complexity of the tool increases (e.g., OneHealth). Explanation of the tool’s methodological foundation in peer-reviewed publications can help experts in countries gain trust for the tools. This trust can then be conveyed to other stakeholders. Repositories of the analyses using the tools may also be helpful. Documentation that describes the tool’s ownership and process of development and validation can further build trust.

For peer review only - http://bmjopen.bmj.com/site/about/guidelines.xhtml

We identified a number of areas where tools can innovate and incorporate outputs that are important for both donors and policymakers. For example, the tools we considered did not make it easy for stakeholders to assess equity. A simple option is to allow disaggregating outputs by subpopulation, and a number of tools already do this; however, tools that do allow disaggregation do not always allow doing this along all dimensions that are important to stakeholders (e.g., wealth). Disaggregating outputs would align with the Disease Control Priorities approach of Extended Cost-Effectiveness Analysis panels.[16] Alternatively, analysts can combine outputs from several runs of a tool using data specific to a given subpopulation each time. However, this puts the onus on tool users (the analysts). Tools could also take novel approaches and help users develop analyses based on distributional cost-effectiveness analysis framework, though data limitations may make this difficult.[17]

An opportunity also exists for building on tools to develop analyses that can compare interventions for different diseases with health system strengthening ones. The OneHealth tool is the most comprehensive among the tools; it incorporates linked modules—which are prioritisation tools themselves focused on specific elements—to be able to make comparisons across diseases and investments to strengthen health systems. That complexity comes at a cost: increased resources from users as well as increased difficulty to validate the consistency of the linked modules, which have been combined over the past two decades and were originally developed by different groups. EQUIST, which includes neonatal, infant, and maternal interventions, takes a completely different approach to health system strengthening. It helps identify bottlenecks and estimates the effect of reducing these bottlenecks on effective coverage. It links to other tools such as LiST and OneHealth to then conduct impact and costing analyses.

For peer review only - http://bmjopen.bmj.com/site/about/guidelines.xhtml

Our study is limited as it involved only desk-based research that included extracting information from guidance documents and peer-reviewed publications and exploring rudimentary analyses using the tools. Further discussion with tool developers and experienced users would provide a more complete understanding of the tools. However, this limitation can also be viewed as an advantage, as it allowed us to understand the tools based on what is available in the public domain. We held a workshop with tool developers and users, but further interviews with them and stakeholders of prioritisation would provide further insight into the challenges of tool development and their future direction.

Overall, we are encouraged by the plethora of different types of tools available for prioritising resource allocation. There is no one tool that is optimal in every case, and different categories of tools are helpful for different parts of the prioritisation process and for different audiences. As outlined in this discussion, we do see gaps and areas in which the tools included in our analysis can be improved.

Acknowledgements: We would like to thank participants of the "Why Good Priority-Setting Tools are More Important for Global Health than Ever Before" workshop, which was held in September 2021. The workshop helped refine the research.

Author contributions:
Concept & design: Megiddo, Ruiz, and Morton
Acquisition of data: Blair and Sabei
Analysis & interpretation: Megiddo, Blair, Sabei, and Morton
Drafting of the manuscript: Megiddo, Blair & Sabei
Critical revision of paper for important intellectual content: Megiddo, Blai, Sabei, Ruiz, and Morton
Provision of study materials: Sabei
Obtaining funding: Megiddo, Ruiz and Morton
Administrative, technical, or logistic support: Ruiz
Supervision: Megiddo and Morton

Competing Interests: All authors received support for this study from Bill & Melinda Gates Foundation through the International Decision Support Initiative. Prof Morton and Dr

For peer review only - http://bmjopen.bmj.com/site/about/guidelines.xhtml

Megiddo reported receiving grants from National Institute of Health Research/ Department of Health and Care Policy Research programme. Prof Morton additionally reported receiving grants from NIHR Global Health Group programme, Chief Scientist's Office Scottish NHS, European Commission, Cancer Research UK, and Pancreatic cancer UK. Prof Morton reported receiving book royalties from Springer. Dr Megiddo additional reported receiving grants from the Scottish Funding Council. Prof Morton reported receiving consulting fees from the Bill & Melinda Gates Foundation via the Thailand Ministry of Public Health and from the WHO Global Malaria Programme. Dr Megiddo reported receiving consulting fees from the International Decision Support Initiative. Prof Morton reported receiving payment or honoraria for lectures from the National University of Singapore. Prof Morton reported participation in the Office of Health Economics board.

Funding: This work received funding support from the Bill and Melinda Gates Foundation (OPP1202541) through the International Decision Support Initiative (iDSI). The funders did not have a role in writing the manuscript or the decision to submit for publication.

Data sharing statement: Data used in the analysis are all publicly available. The Supplementary Materials lists and links to the documentation used in the analysis.

Ethics statements:

Patient consent for publication: not applicable

Ethical approval: not applicable

For peer review only

References

1. Wilkinson T, Sculpher MJ, Claxton K, et al. The International Decision Support Initiative Reference Case for Economic Evaluation: An Aid to Thought. *Value in Health*. 2016;19(8):921-928. doi:10.1016/j.jval.2016.04.015

2. Glassman A, Chalkidou K, Giedion U, et al. Priority-Setting Institutions in Health: Recommendations from a Center for Global Development Working Group. *Global Heart*. 2012;7(1):13-34. doi:10.1016/j.gheart.2012.01.007

3. Airoldi M, Morton A, Smith JAE, Bevan G. STAR—People-Powered Prioritization: A 21st-Century Solution to Allocation Headaches. *Med Decis Making*. 2014;34(8):965-975. doi:10.1177/0272989X14546376

4. Mitton C, Donaldson C, Dean S, West B. Program budgeting and marginal analysis: a priority-setting framework for Canadian Regional Health Authorities. *Healthc Manage Forum*. 2000;13(4):24-31. doi:10.1016/S0840-4704(10)60790-9

5. Daniels N, Sabin JE. Accountability for reasonableness: an update. *BMJ*. 2008;337:a1850. doi:10.1136/bmj.a1850

6. Baltussen R, Jansen MP, Mikkelsen E, et al. Priority Setting for Universal Health Coverage: We Need Evidence-Informed Deliberative Processes, Not Just More Evidence on Cost-Effectiveness. *Int J Health Policy Manag*. 2016;5(11):615-618. doi:10.15171/ijhpm.2016.83

7. The Global Fund to Fight AIDS, Tuberculosis and Malaria. *Funding Request Instructions*. The Global Fund to Fight AIDS, Tuberculosis and Malaria; 2020. Accessed September 20, 2021. https://www.theglobalfund.org/media/5743/fundingrequest_fullreview_instructions_en.pdf

8. Delva W, Wilson DP, Abu-Raddad L, et al. HIV Treatment as Prevention: Principles of Good HIV Epidemiology Modelling for Public Health Decision-Making in All Modes of Prevention and Evaluation. *PLoS Med*. 2012;9(7). doi:10.1371/journal.pmed.1001239

9. Clapham H, Gad M, Gheorghe A, et al. Assessing fitness-for-purpose and comparing the suitability of COVID-19 multi-country models for local contexts and users. *Gates Open Res*. 2021;5:79. doi:10.12688/gatesopenres.13224.1

10. Policy Group, Covid-19 Multi-Model Collaboration (CMCC). *Guidance on Use of Modelling for Policy Responses to COVID-19*. CMCC; 2020. Accessed August 26, 2020. https://decidehealth.world/sites/default/files/2020-08/CMCC%20Policy%20Group%20Report%20August%202020.pdf

11. The Global Fund. The Global Fund | The Data Explorer - Documents. Accessed April 13, 2023. https://data.theglobalfund.org/documents

12. Global Financing Facility. Resource Library | Global Financing Facility. Accessed April 13, 2023. https://www.globalfinancingfacility.org/resource-library

For peer review only - http://bmjopen.bmj.com/site/about/guidelines.xhtml

13. McCormick BJJ, Waiswa P, Nalwadda C, Sewankambo NK, Knobler SL. SMART Vaccines 2.0 decision-support platform: a tool to facilitate and promote priority setting for sustainable vaccination in resource-limited settings. *BMJ Global Health*. 2020;5(11):e003587. doi:10.1136/bmjgh-2020-003587

14. Optima Consortium for Decision Science. Optima HIV User Guide Vol. VI: Parameter Data Sources. Published online 2021. Accessed September 6, 2022. https://docs.google.com/document/d/1VIxB08GjnLhUjRwLAKuBJ-To2WXud7krK9CNNu6NwIg/edit?usp=drive_open&ouid=106214940111356882136&usp=embed_facebook

15. Fraser-Hurt N, Hou X, Wilkinson T, et al. Using allocative efficiency analysis to inform health benefits package design for progressing towards Universal Health Coverage: Proof-of-concept studies in countries seeking decision support. *PLOS ONE*. 2021;16(11):e0260247. doi:10.1371/journal.pone.0260247

16. Jamison DT, Gelband H, Horton S, et al., eds. *Disease Control Priorities: Improving Health and Reducing Poverty*. Third edition. World Bank Group; 2018.

17. Cookson R, Griffin S, Norheim OF, Culyer AJ, eds. *Distributional Cost-Effectiveness Analysis: Quantifying Health Equity Impacts and Trade-Offs*. Oxford University Press; 2020. Accessed September 23, 2021. https://oxfordmedicine.com/view/10.1093/med/9780198838197.001.0001/med-9780198838197

For peer review only

Supplementary Material 1. Tool documentation

CAPACITI (Country-led Assessment for Prioritization in Immunization)
Guidance manual

PriorityVax
User guide
Phase 1 report
Phase 2 report
Phase 3 report

Optima
HIV User Guide
TB User Guide
HIV allocative efficiency analysis training workshop agenda and training materials
TB allocative efficiency analysis training workshop agenda and training materials
Software Reference Manual
Expenditure Data Guide
HIV-related Abbreviations
Parameter Data Sources

OneHealth
FAQ
Startup Manual
Technical Notes
Technical Notes Annexes
Intervention Input Assumptions Manual
Defining the target population PIN and coverage in OneHealth
Using OneHealth for programme planning
MNS Module Manual
Manual

LiST (Lives Saved Tool)
Manual
Subnational modeling
Nutrition modeling in LiST

EQUIST (Equitable strategies to save lives)
Technical notes
User guide

For peer review only

Supplementary materials 2. Tables

Table 1. Tools additional information

	Year created	Owned / developed by	Time/process to implement	Required level of technical expertise
CAPACITI	2020	WHO	A typical recommendation is expected to take from 4 to 6 months. Intensive data collection required.<br><br>1. Clarify decision question<br>2. Set criteria for decision-making<br>3. Collect and assess evidence<br>4. Appraisal of results<br>5. Finalisation and communication of recommendation	Minimal (No/minimal training is required)
PriorityVax	2010**	Sabin Vaccine Institute	No information given on timing of process.<br><br>1. Policy questions are defined (Define decision criteria, Discuss and identify attributes, Explicitly weight criteria, Discuss and weight attributes)<br>2. PICO questions are defined (Population, Intervention, Comparator, Outcome)<br>3. Evidence gathered and reviewed to address PICO questions and evidence attributes<br>4. Evidence used to extract values and/or score attributes<br>5. Recommendation provided<br>6. Interactive sensitivity analysis	Minimal (No/minimal training is required)
HIPtool	NA	WB	No formal information on process, intensive data collection required.	Minimal (No/minimal training is required)
Optima	2004	OPTIMA consortium	No formal information on process, intensive data collection required.	Detailed knowledge required (3 days intensive training)
OneHealth (Spectrum suite)	1997	Avenir Health	A typical recommendation is expected to take from 4 to 6 months:<br><br>1. Initiation of planning process.<br>2. Stakeholder meeting (first plenary).<br>3. Tool set-up with baseline data entry.<br>4. Second plenary.<br>5. Individual modules/groups.<br>6. Assessing the joint plan.<br>7. Adjusting individual modules/groups.<br>8. Submission of revised modules.<br>9. Group discussion and final presentation of NHSP (National health service plan)	Detailed knowledge required
LiST	2003	Institute for International Programs at Johns Hopkins Bloomberg School of Public Health	No information in user manual or technical notes on likely process and timelines for implementation	Minimal (No/minimal training is required)
EQUIST	2016	UNICEF in association with Johns Hopkins Bloomberg School of Public Health and WB	No information given on timing of process.<br><br>Users conduct a situational analysis, comprising an equity profile analysis and an equity frontier analysis, followed by a scenario analysis, as follows:<br><br>1. Define priority / target populations<br>2. Prioritise epidemiological priorities<br>3. Prioritise interventions<br>4. Prioritise key bottlenecks	Minimal (No/minimal training is required)

For peer review only

			5. Prioritise causes of bottlenecks<br>6. Select strategies to address cause of bottleneck<br>7. Assess expected impact and cost effectiveness	

CAPACITI: Country-led Assessment for Priortization in Immunization; HIPtool: Health Interventions Priortization tool; LiST: Lives Saved Tool; EQUIST: Equitable strategies to save lives; LMIC: Low- and Middle-Income Country; MNCH&N: Maternal, Newborn, Child Health and Nutrition.
* Adaptability to extend to new diseases and interventions.
** The Institute of Medicine commissioned the tool in 2010, and it was developed with the name SmartVaccines. This evolved into PriorityVax by 2018.

For peer review only
